# Effects of tumor-specific CAP1 expression and body constitution on clinical outcomes in patients with early breast cancer

**DOI:** 10.1186/s13058-020-01307-5

**Published:** 2020-06-19

**Authors:** Malin Bergqvist, Karin Elebro, Malte Sandsveden, Signe Borgquist, Ann H. Rosendahl

**Affiliations:** 1 Department of Clinical Sciences Lund, Oncology, Skåne University Hospital, Lund University, Lund, Sweden; 2 Department of Clinical Sciences Malmö, Surgery, Skåne University Hospital, Lund University, Malmö, Sweden; 3 Department of Oncology, Clinical Medicine, Aarhus University Hospital, Aarhus University, Aarhus, Denmark

**Keywords:** Breast cancer, CAP1, Obesity, Survival, Prognosis

## Abstract

**Background:**

Obesity induces molecular changes that may favor tumor progression and metastatic spread, leading to impaired survival outcomes in breast cancer. Adenylate cyclase-associated protein 1 (CAP1), an actin regulatory protein and functional receptor for the obesity-associated adipokine resistin, has been implicated with inferior cancer prognosis. Here, the objective was to investigate the interplay between body composition and CAP1 tumor expression regarding breast cancer outcome through long-term survival analyses.

**Methods:**

Among 718 women with primary invasive breast cancer within the large population-based prospective Malmö Diet and Cancer Study, tumor-specific CAP1 levels were assessed following thorough antibody validation and immunohistochemical staining of tumor tissue microarrays. Antibody specificity and functional application validity were determined by *CAP1* gene silencing, qRT-PCR, Western immunoblotting, and cell microarray immunostaining. Kaplan-Meier and multivariable Cox proportional hazard models were used to assess survival differences in terms of breast cancer-specific survival (BCSS) and overall survival (OS) according to body composition and CAP1 expression.

**Results:**

Study participants were followed for up to 25 years (median 10.9 years), during which 239 deaths were observed. Patients with low CAP1 tumor expression were older at diagnosis, displayed anthropometric measurements indicating a higher adiposity status (wider waist and hip, higher body mass index and body fat percentage), and were more prone to have unfavorable tumor characteristics (higher histological grade, higher Ki67, and estrogen receptor (ER) negativity). Overall, patients with CAP1-low tumors had impaired BCSS (adjusted hazard ratio: HR_adj_ = 0.52, 95% CI 0.31–0.88) and OS (HR_adj_ = 0.64, 95% CI 0.44–0.92) compared with patients having high CAP1 tumor expression. Further, analyses stratified according to different anthropometric measures or ER status showed that the CAP1-associated survival outcomes were most pronounced among patients with low adiposity status or ER-positive disease.

**Conclusions:**

Low CAP1 tumor expression was associated with higher body fatness and worse survival outcomes in breast cancer patients with effect modification by adiposity and ER status. CAP1 could be a novel marker for poorer survival outcome in leaner or ER-positive breast cancer patients, highlighting the need for considering body constitution in clinical decision making.

## Introduction

Breast cancer is the most common female malignancy worldwide with around two million new diagnoses annually [1]. With the introduction of improved diagnostic and treatment modalities, survival has improved significantly, with 5- and 10-year survival rates in Sweden reaching 90% and 81%, respectively [2]. However, breast cancer is still a leading cause of cancer-related mortality in women, due to progressive disease with distant metastases [1].

Obesity is an established risk factor for several types of cancer and is expected to supersede smoking as the dominant cause of cancer in the near future [3, 4]. The steady increasing prevalence of obesity constitutes a global health concern associated with an increasing breast cancer incidence [5, 6]. Breast cancer patients with a higher body mass index (BMI) are further more likely to have larger tumor size at diagnosis and to develop distant metastasis with worse prognosis [7, 8]. Obesity is frequently linked to metabolic complications, such as insulin resistance and systemic low-grade inflammation, which may favor a pro-tumorigenic environment [9]. The adipose tissue, now recognized as an endocrine organ, secretes local and systemic bioactive adipokines with implications for tumor development [10]. Among these adipokines, circulating resistin levels have been reported to be positively correlated with obesity and incidence of postmenopausal breast cancer [11–14]. In line with this, we previously demonstrated enhanced secretion of resistin by adipocytes during obesity-related conditions in a preclinical study [15]. Clinically, high levels of resistin in breast cancer tissue have been linked to more advanced tumor stage with large tumor size and lymph node involvement, positive estrogen receptor (ER) status, and poor breast cancer outcome [16].

It was recently discovered that resistin interacts with adenylate cyclase-associated protein 1 (CAP1), a highly conserved actin-binding protein involved in cytoskeletal rearrangements [17, 18]. CAP1 is ubiquitously expressed in most tissues while its homolog CAP2 appears confined to brain and muscle tissue [19, 20]. CAP1 is a multi-domain protein that localizes to the dynamic regions of the cortical actin cytoskeleton where it promotes cofilin-induced actin filament depolymerization and contributes to rapid actin turnover [21]. Spatial and temporal actin dynamics are a necessity for cytoskeletal rearrangements and formation of membrane protrusions required for tumor cell motility, invasiveness, and metastatic dissemination [22]. Understanding these biological processes is vital to prevent breast cancer progression and metastatic dissemination. Under obese conditions, the adipocyte secretome has been shown to stimulate membrane protrusions and motility in CAP1-expressing breast cancer cells [15]. CAP1 has additionally been reported involved in tumorigenic processes such as cell cycle regulation, proliferation, and adhesion [23–25]. While high *CAP1* gene expression has been linked to poor tumor characteristics and worse breast cancer prognosis, associations between CAP1 protein expression and body constitution and clinical outcome in breast cancer are is yet unknown.

The aim of this study was to evaluate whether CAP1 tumor expression was associated to body constitution and clinical outcome in breast cancer. Based on previous cellular and gene expression studies, our hypotheses were that an obese body composition would be associated with high CAP1 expression in tumors and that breast cancer patients with high CAP1 tumor expression would have worse prognosis. In order to test this, we assessed tumor-specific CAP1 protein expression and anthropometric measures in a cohort of 1016 patients with incident breast cancer and long-term follow-up within the prospective population-based Malmö Diet and Cancer Study (MDCS).

## Material and methods

### The Malmö Diet and Cancer Study

The MDCS enrolled participants living in Malmö, Sweden, between 1991 and 1996 with the objective to explore associations between dietary habits and subsequent cancer risk. This prospective population-based cohort included 17,035 women born 1923–1950, representing 42.6% of the eligible population [26, 27]. Exclusion criteria were limited to Swedish language insufficiency and mental disabilities impairing the respondent’s completion of study questionnaires. At baseline, the participants answered extensive questionnaires, underwent anthropometric measures including height, weight, waist and hip circumference, and bioelectrical impedance analysis of body fat percentage (BF%) obtained by trained study nurses, and blood samples were collected. Of the 17,035 study participants, 576 had a prevalent breast cancer diagnosis prior to baseline examination and were thus excluded. Information on incident breast cancer cases and vital status has been retrieved annually through record linkage to the Swedish Cancer Registry, the Southern Swedish Regional Tumor Registry, and the Swedish Cause of Death Registry [27]. Ethical approval was obtained from the Ethical Committee at Lund University (Dnr 427/2007) and all study participants signed informed consent at enrollment.

### Study population

**Fig. 1:**
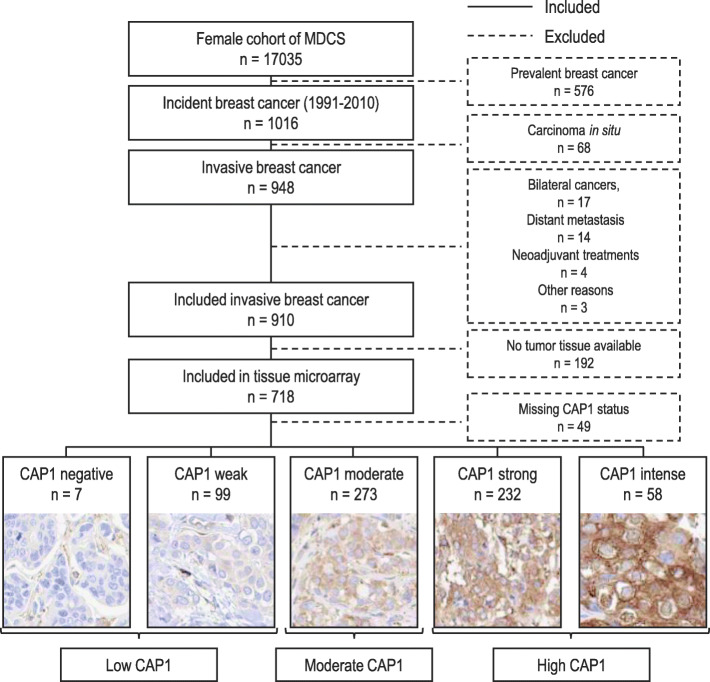
Flowchart of the study population from the Malmö Diet and Cancer Study (MDCS)

In total, 1016 women were diagnosed with primary breast cancer prior to January 1, 2011. Patients with carcinoma in situ only (*n* = 68), bilateral cancers (*n* = 17), distant metastasis at diagnosis (*n* = 14), neoadjuvant treatment (*n* = 4), breast cancer-related death within 0.3 years from diagnosis (*n* = 2), and one patient who declined treatment for 4 years prior to accepting surgery (*n* = 1) were excluded from the study population. The remaining study population consisted of 910 patients with incident invasive breast cancer of whom 718 had tumor tissue available in a prepared tissue microarray (TMA). A flowchart of the study population is shown in Fig. 1.

### Clinical and pathology information

Information on tumor characteristics and breast cancer treatments were retrieved from medical records. For patients diagnosed prior to 2005, histological tumor type and grade were re-evaluated according to the WHO and Nottingham classifications [28] by one senior breast pathologist [29].

Information on tumor markers were retrieved from medical records and from immunohistochemistry (IHC) assessments of tumor tissue microarray (TMA) at the Center for Molecular Pathology, Malmö University Hospital, Malmö, Sweden, as previously described [29, 30]. In brief, estrogen receptor (ER) and progesterone receptor (PR) status were obtained from TMA data (1991–2004) and from medical records (2005 onward). Human epidermal growth factor receptor 2 (HER2) status was retrieved from TMA data, medical records, and patient registers (1991–2004) and medical records and patient registers (2005 onward). Ki67 proliferation index was obtained from TMA data (1991–2007) and from medical records (2008 onward).

In accordance with the Swedish clinical guidelines, ER and PR status were considered positive if > 10% of cancer cell nuclei were stained. Ki67 positivity was categorized into three groups (low, intermediate and high) based on tertile distribution within three assessment periods: 1991–2004, 2005–2007, and 2008–2014 [31]. HER2 was primary classified by in situ hybridization (ISH) and secondly by IHC. HER2 was considered positive if ISH showed HER2 amplification or if IHC was graded 3+. When ISH was negative for HER2 amplification or IHC was annotated 1+ or less, the tumor was regarded as HER2-negative. If no ISH data existed and IHC was graded 2+, HER2 status was regarded as missing.

### Classification of breast cancer subtypes

Surrogate molecular subtypes were created based on ER, PR, and HER2 receptor status along with Ki67 positivity and histologic grade [31, 32]. Tumors were classified into *luminal A-like* [ER+ and HER2− tumors with (a) histologic grade I or (b) histologic grade II and low Ki67 or (c) histologic grade II, intermediate Ki67 and PR+], *luminal B-like* [ER+ and HER2− tumors with (a) histologic grade III or (b) histologic grade II and high Ki67 or (c) histologic grade II, intermediate Ki67 and PR−], *HER2-positive* [all HER2+ tumors], or *triple-negative* [all ER−, PR−, HER2− tumors].

### Cell culture

Antibody validation for IHC assessment of CAP1 was performed using established breast cancer cell models. ER-positive T47D and ER-negative MDA-MB-231 human breast cancer cell lines were purchased and validated by ATCC-LGC Standards. The cells were cultivated in Dulbecco’s modified Eagle’s medium (DMEM; with GlutaMAX™ and HEPES) supplemented with antibiotics (100 U/ml penicillin and 100 μg/ml streptomycin) and 10% fetal bovine serum. The cells were grown in a humidified 5% CO_2_ atmosphere at 37 °C, routinely passaged once a week.

### siRNA knockdown

Knockdown of *CAP1* in breast cancer cells was obtained using small interfering RNA (siRNA) with reverse transfection. Three different siRNA constructs (*Silencer*® Select s20547, s20548, s20579, ThermoFisher Scientific) were tested alone or in combination for optimal target knockdown. Briefly, the final siRNA transfection was performed as follows: 0.55 × 10^6^ of cells in 1.25 ml of antibiotics-free cell culture medium were added to a mixture of 25 nM CAP1 siRNA (T47D: s20547 and s20549; MDA-MB-231: s20549) and 10 μl Lipofectamine 2000 in 1.25 ml OptiMEM in 6-well plates. The *Silencer®* Select Negative Control No1 siRNA (ThermoFisher Scientific) was used as a non-targeting control. Following 72 h incubation, the cells were washed and collected for further analyses.

### Quantitative reverse transcription PCR

Total RNA was extracted using QIAGEN RNeasy (Qiagen, Mississauga, ON, Canada) according to the manufacturer’s instructions and quantified via Qubit Fluorometric system (Thermo Scientific, Waltham, MA, USA). cDNA was synthesized from 1 μg of total RNA using High Capacity cDNA Reverse Transcription kit (Thermo Scientific, Waltham, MA, USA). Quantitative reverse transcription PCR (qRT-PCR) was performed using TaqMan QuantiTect Probe kit (Qiagen, Mississauga, ON, Canada) with primers specific for *CAP1* (Hs02860542_g1 ThermoFisher Scientific, Waltham, MA, USA). *GAPDH* (Hs99999905_m1 ThermoFisher Scientific, Waltham, MA, USA) was used as reference gene. All transcripts were measured in minimum duplicates and normalized to *GAPDH*. Relative CAP1 mRNA expression levels in *CAP1* silenced cells compared with control were determined by 2−ΔΔCt method in three independent experiments (Additional file 1A).

### Western immunoblotting

Breast cancer cell lysates were prepared from CAP1 knockdown or control cells, and proteins were extracted using radioimmunoprecipitation assay buffer [RIPA; 10 mM Tris-HCl pH 7.4, 50 mM NaCl, 5 mM EDTA, 30 mM sodium pyrophosphate, 50 mM sodium fluoride, 100 μM sodium orthovanadate, 1% Triton X-100] supplemented with protease and phosphatase inhibitors. Protein quantifications were performed using Pierce BCA Protein Assay Kit, according to the manufacturer’s instructions. Proteins were separated by pre-cast SDS-PAGE (NuPAGE 10% Bis-Tris, Invitrogen) and transferred to nitrocellulose membrane. The membrane was blocked with 5% (w/v) non-fat dry milk in Tris-buffered saline with Tween-20 and incubated overnight at 4 °C with primary antibodies to CAP1 (Abcam; ab133655, 1:10000) or GAPDH (Merck; MAB374, 1:1000). The blots were subsequently incubated with horseradish peroxidase-conjugated secondary antibodies (CAP1, 1:2000; GAPDH, 1:10000) for 1 h and proteins visualized using SuperSignal West Dura Extended Duration Substrate (ThermoFisher Scientific) and LI-COR Biosciences Odyssey Imaging System. Relative protein levels were quantified by densitometry using ImageJ software (NIH) and normalized against the GAPDH housekeeping protein.

### Cell microarray and immunocytochemistry of CAP1 knockdown cells

Cell pellets of T47D and MDA-MB-231 breast cancer cells collected following siRNA exposure were fixed in 4% formalin overnight, stained with hematoxylin, dehydrated, and paraffin embedded. A cell microarray was constructed with multiple 1.0 mm cores from each cell preparation using a semi-automatic Tissue Arrayer (Pathology Devices, MD, USA). For immunocytochemistry analysis, 3 μm sections were automatically pretreated using a pressure cooker (Histolab Products AB) and stained for CAP1 (Abcam; ab133655, 1:10,000) using Autostainer Plus (Agilent, DK).

### Immunohistochemical staining and CAP1 evaluation

Following antibody validation, immunohistochemical staining of tumor-specific CAP1 was performed using a prepared TMA with duplicate 1 mm tissue cores from individual tumors of the 718 patients in the study population with available tumor tissue (Fig. 1). TMA sections (4 μm) were automatically deparaffinized and pretreated for antigen retrieval using a pressure cooker (Histolab Products AB). IHC was performed and stained for CAP1 (Abcam; ab133655 at 1:10,000 for 30 min) using EnVision FLEX, high pH (Agilent K801021-2) and Autostainer Plus (Agilent, Denmark) with subsequent hematoxylin counterstaining (Agilent S2020).

Evaluation of the CAP1 staining intensity was done twice by one observer (MB), blinded to patient information and tumor characteristics, using light microscope Olympus BX53. The overall CAP1 protein positivity rate was > 75% in tumor cells, whereby only differences in CAP1 staining intensity was annotated. Cytoplasmic CAP1 staining intensity was assessed in five categories: negative (−), weak (1+), moderate (2+), strong (3+), and intense (4+), with representative images of staining intensities shown in Fig. 1. Conflicting assessments were low (4% of the cases), and in cases of discrepancy between the readings, a third evaluation was made. For remaining inconsistency, a second observer (AR) was consulted until consensus. In the event of intra- or inter-duplicate tumor core heterogeneity, the highest score was applied for tumors borderline between two scores. Based on the distribution of CAP1 staining intensities and survival estimates in relation to the initial five CAP1 categories (Additional file 2), the CAP1 tumor expression were subsequently compiled into three groups according to the cytoplasmic intensity scores: low [negative (−)/weak (1+)], moderate [moderate (2+)], and high [strong (3+)/intense (4+)].

### Statistical analysis

The difference in distribution by CAP1 expression across patient characteristics or clinicopathological parameters was analyzed by linear-by-linear association chi-square test for trend for the categorical data and Jonckheere-Terpstra for the comparisons of medians. Categorical variables are presented as number (*n*) and frequency (%) of patients, continuous variables as medians with interquartile range (IQR). In survival analyses, Kaplan-Meier estimates and LogRank-trend test were used to assess the association between CAP1 expression and time-to-event defined as breast cancer-specific survival (BCSS) or overall survival (OS). The follow-up time was defined as date of diagnosis to date of death, emigration, or end of follow-up up until December 31, 2016. Univariable and multivariable Cox regression models were used to calculate crude and adjusted hazard ratios (HRs) with 95% confidence intervals (CI) for association between CAP1 tumor expression and breast cancer outcome. Models were adjusted for potential confounding factors. Model 1 was unadjusted (crude), and model 2 was adjusted for age at diagnosis (continuous), tumor size (≤ 20 mm or > 20 mm) and any lymph node involvement (positive or negative). Model 3 was additionally adjusted for histologic grade (1–2 or 3), Ki67 (low, intermediate or high, HER2 (normal or overexpression), and ER (positive or negative). Student’s *t* test was applied to test statistical difference between two groups for experimental in vitro data. Statistical analyses were performed using IBM SPSS Statistics for Windows v 25.0 (Armonk, NY: IBM Corp.) for clinical data and GraphPad Prism v 7.03 for experimental data. All tests were two-tailed, and the *P* value was considered as strength of evidence against the null hypothesis. The study adheres to the REporting recommendations for tumor MARKer prognostic studies (REMARK) guidelines, to ensure methodological quality [33].

## Results

### CAP1 antibody validation

To ensure target specificity and functional application validity, a thorough antibody validation was performed prior to IHC staining. The specificity of the antibody was determined by applying a genetic strategy of siRNA-mediated *CAP1* gene knockdown. qRT-PCR analyses confirmed an efficient *CAP1* knockdown with 6 and 2% detectable mRNA expression in T47D and MDA-MB-231 cells, respectively, compared with the non-silencing controls (all *P* values < 0.001, Additional file 1A). The specificity and functional application of the monoclonal Abcam CAP1 antibody was further determined by Western immunoblotting and immunocytochemistry of constructed cell microarray. Equivalent to the mRNA results, approximately 90% and > 99% reduction of CAP1 protein expression at the expected molecular size (52 kDa) was detected by the Abcam antibody in the CAP1 silenced T47D and MDA-MB-231 cells, respectively (all *P* values < 0.001, Additional file 1B). Similar findings were obtained in the immunocytochemistry analyses (Additional file 1C), thereby confirming the validity of the antibody. An independent antibody validation was performed using an affinity isolated polyclonal CAP1 Prestige antibody (Sigma Aldrich; HPA030124), with equivalent results (data not shown).

### Patient characteristics and tumor-specific CAP1 protein expression

Among the 718 breast cancer patients with tumor tissue included in the TMA, CAP1 expression was assessable in 669 tumors (Fig. 1), of which 106 tumors (15.8%) displayed a low, 273 (40.8%) moderate, and 290 (43.3%) high CAP1 expression. Baseline patient characteristics according to distribution of CAP1 intensities are presented in Table 1. Patients with tumors of low to moderate CAP1 expression were more likely to be older at baseline (*P* < 0.001), have a higher BMI (*P* = 0.009), larger waist (*P* = 0.008), wider hip (*P =* 0.014), and higher BF% (*P* = 0.006), compared to patients with tumors of higher CAP1 expressions. No further associations were found between CAP1 expression and anthropometric measures.

**Table 1:** Distribution of patient characteristics and CAP1 expression

		Patients with tumor in TMA (*n* = 718)					
Patient characteristics *n* (%) or median (IQR)	All patients (*n* = 910)	Low CAP1 (*n* = 106)	Moderate CAP1 (*n* = 273)	High CAP1 (*n* = 290)	*P*_trend_	Non-assessable (*n* = 49)	Not included in TMA (*n* = 192)
**Age at baseline**							
Continuous [years]	55.4 (50.0–61.7)	56.2 (51.9–62.2)	56.2 (50.6–61.7)	53.5 (49.0–59.4)	**< 0.001**^**a**^	52.3 (48.2–57.4)	58.2 (51.7–62.3)
**Menopause**							
Premenopausal	68 (7.9)	4 (3.9)	21 (8.0)	23 (8.3)	0.216^b^	5 (11.1)	15 (8.5)
Postmenopausal	792 (92.1)	98 (96.1)	240 (92.0)	253 (91.7)		40 (88.9)	161 (91.5)
Missing	50	4	12	14		4	16
**Height**							
Continuous [cm]	165.0 (160.0–168.0)	164.5 (161.0–167.3)	164.0 (160.0–168.0)	165.0 (161.0–169.0)	0.091^a^	163.0 (160.0–167.5)	165.0 (161.0–168.0)
**Weight**							
Continuous [kg]	68.0 (61.0–75.0)	69.0 (60.8–77.3)	69.0 (62.0–75.0)	67.0 (61.0–74.0)	0.060^a^	65.0 (61.0–73.5)	68.0 (62.3–75.0)
**BMI**							
Continuous [kg/m^2^]	24.8 (22.8–27.8)	25.1 (22.5–28.6)	25.3 (23.1–28.5)	24.4 (22.2–27.4)	**0.009**^**a**^	24.3 (22.8–27.6)	24.7 (22.9–27.5)
< 25	466 (51.2)	52 (49.0)	126 (46.2)	161 (55.5)	0.053^b^	30 (61.2)	97 (50.5)
25–30	310 (34.1)	34 (32.1)	101 (37.0)	91 (31.4)		13 (26.5)	71 (37.0)
> 30	134 (14.7)	20 (18.9)	46 (16.9)	38 (13.1)		6 (12.2)	24 (12.5)
**Waist**							
Continuous [cm]	77.0 (71.0–83.0)	77.0 (71.0–85.3)	78.0 (72.0–86.0)	76.0 (70.0–83.0)	**0.008**^**a**^	76.0 (69.5–83.0)	76.5 (71.0–82.0)
< 81	603 (66.3)	64 (60.4)	171 (62.6)	197 (68.2)	0.063^b^	33 (67.3)	138 (71.9)
81–85	116 (12.8)	16 (15.1)	30 (11.0)	43 (14.9)		7 (14.3)	20 (10.4)
> 85	190 (20.9)	26 (24.5)	72 (26.4)	49 (16.9)		9 (18.4)	34 (17.7)
Missing	1	0	0	1		0	0
**Hip**							
Continuous [cm]	97.0 (91.0–103.0)	98.0 (92.0–106.0)	97.0 (91.0–104.0)	96.0 (90.0–102.0)	**0.014**^**a**^	95.0 (90.0–103.0)	97.0 (92.0–102.8)
Missing	1	0	0	1		0	0
**Waist-hip ratio**							
Continuous	0.79 (0.76–0.83)	0.79 (0.76–0.83)	0.80 (0.77–0.83)	0.79 (0.76–0.82)	0.095^a^	0.79 (0.75–0.84)	0.78 (0.75–0.82)
≤ 0.80	522 (57.4)	63 (59.4)	135 (49.5)	175 (60.6)	0.810^b^	27 (55.1)	122 (63.5)
0.81–0.84	268 (29.5)	33 (31.1)	100 (36.6)	73 (25.3)		14 (28.6)	48 (25.0)
≥ 0.85	119 (13.1)	10 (9.4)	38 (13.9)	41 (14.2)		8 (16.3)	22 (11.5)
Missing	1	0	0	1		0	0
**Body fat percentage**							
Continuous [%]	31.0 (27.8–34.0)	31.5 (28.0–35.0)	32.0 (28.0–35.0)	30.0 (27.0–33.8)	**0.006**^**a**^	30.0 (26.0–34.0)	31.0 (28.0–34.0)
≤ 24	83 (10.3)	9 (8.5)	20 (7.4)	46 (16.0)	**0.007**^**b**^	5 (10.2)	13 (6.8)
25–31	395 (43.6)	44 (41.5)	114 (42.1)	121 (42.0)		23 (46.9)	93 (48.4)
≥ 32	418 (46.1)	53 (50.0)	137 (50.6)	121 (42.0)		21 (42.9)	86 (44.8)
Missing	4	0	2	2		0	0

^a^Jonckheere-Terpstra test

^b^Linear-by-linear association test. *P* value < 0.05 in bold

### Distribution of clinicopathological characteristics and CAP1 tumor expression

Patients with low to moderate CAP1-expressing tumors were older at breast cancer diagnosis (*P* < 0.001) compared to patients with tumors of high CAP1 expressions (Table 2). Further, patients with low CAP1-expressing tumors were more likely to present with high proliferative (defined as Ki67 high; *P* < 0.001) and histological grade III (*P* < 0.001) tumors and had a higher percentage of ER-negative tumors than patients with tumors of higher CAP1 intensities (*P =* 0.014). Similarly, low CAP1 expression was more frequent among patients with HER2-positive and triple-negative (*P* < 0.001) molecular subtypes. Patients with low CAP1 tumor expression tended to be more likely to received adjuvant endocrine treatment compared with patients with moderate to high CAP1 tumor expression (*P* = 0.002; Additional file 3). Other breast cancer treatment modalities were not related to CAP1 tumor expression.

**Table 2:** Distribution of tumor characteristics and CAP1 tumor-specific expression

		Patients with tumor in TMA (*n* = 718)					
Patient characteristics	All patients	Low CAP1	Moderate CAP1	High CAP1	*P*_trend_	Non-assessable	Not included
*n* (%) or median (IQR)	(*n* = 910)	(*n* = 106)	(*n* = 273)	(*n* = 290)		(*n* = 49)	in TMA (*n* = 192)
**Age at diagnosis**							
Continuous [years]	65.0 (60.0–71.6)	65.4 (60.8–73.3)	66.6 (61.4–72.3)	63.9 (58.8–69.5)	**< 0.001**^**a**^	60.7 (55.0–66.4)	65.6 (60.4–71.9)
**Tumor size [mm]**							
≤ 20	637 (71.8)	64 (60.4)	189 (69.5)	205 (71.2)	0.066^b^	40 (83.3)	139 (80.3)
> 20	250 (28.2)	42 (39.6)	83 (30.5)	83 (28.8)		8 (16.7)	34 (19.7)
Missing	19	0	0	0		0	19
**Axillary node involvement**							
Negative	557 (68.0)	65 (63.7)	168 (63.9)	181 (67.0)	0.453^b^	36 (80.0)	107 (77.0)
Positive (≥ 1)	262 (32.0)	37 (36.3)	95 (36.1)	89 (33.0)		9 (20.0)	32 (23.0)
Missing	53	0	0	0		0	53
**Histological grade**							
I	227 (27.2)	12 (11.4)	55 (20.6)	99 (34.5)	**< 0.001**^**b**^	14 (33.3)	47 (35.1)
II	392 (46.9)	48 (45.7)	128 (47.9)	138 (48.1)		17 (40.5)	61 (45.5)
III	216 (25.9)	45 (42.9)	84 (31.5)	50 (17.4)		11 (26.2)	26 (19.4)
Missing	75	1	6	3		7	58
**Ki67 status**							
Low	258 (40.7)	27 (30.7)	62 (29.2)	118 (54.4)	**< 0.001**^**b**^	11 (39.3)	40 (45.5)
Intermediate	196 (31.0)	26 (29.5)	76 (35.8)	65 (30.0)		6 (21.4)	23 (26.1)
High	179 (28.3)	35 (39.8)	74 (34.9)	34 (15.7)		11 (39.3)	25 (28.4)
Missing	277	18	61	73		21	104
**ER status**							
Positive (> 10%)	701 (88.8)	79 (79.0)	235 (90.0)	246 (90.1)	**0.014**^**b**^	31 (81.6)	110 (94.0)
Negative (≤ 10%)	88 (11.2)	21 (21.0)	26 (10.0)	27 (9.9)		7 (18.4)	7 (6.0)
Missing	121	6	12	17		11	75
**PR status**							
Positive (> 10%)	438 (58.2)	47 (49.0)	165 (65.7)	147 (56.5)	0.729^b^	19 (50.0)	60 (56.1)
Negative (≤ 10%)	314 (41.8)	49 (51.0)	86 (34.3)	113 (43.5)		19 (50.0)	47 (43.9)
Missing	158	10	22	30		11	85
**HER2 status**							
HER2+	65 (9.2)	14 (16.9)	19 (7.9)	21 (8.3)	0.071^b^	3 (9.1)	8 (8.1)
HER2−	645 (90.8)	69 (83.1)	223 (92.1)	232 (91.7)		30 (90.9)	91 (91.9)
Missing	200	23	31	37		16	93
**Molecular subtypes**							
Luminal A-like	352 (55.4)	29 (37.2)	110 (49.3)	148 (66.4)	**< 0.001**^**b**^	13 (46.4)	52 (62.7)
Luminal B-like	159 (25.0)	21 (26.9)	70 (31.4)	38 (17.0)		9 (32.1)	21 (25.3)
HER2+	65 (10.2)	14 (17.9)	19 (8.5)	21 (9.4)		3 (10.7)	8 (9.6)
Triple-negative	59 (9.3)	14 (17.9)	24 (10.8)	16 (7.2)		3 (10.7)	2 (2.4)
Missing	275	28	50	67		21	109

^a^Jonckheere-Terpstra test

^b^Linear-by-linear association test. *P* value < 0.05 in bold

### Clinical outcome by CAP1 tumor expression in relation to ER status

Patients were followed for up to 25 years, with a median follow-up time of 10.9 years. The median time between baseline examination and date of breast cancer diagnosis were 9.6 years. Of the patients included in the survival analyses, 239 died during follow-up, and 117 of these died from breast cancer-related causes. Three patients had emigrated, and 476 were still alive at end of follow-up.

**Fig. 2:**
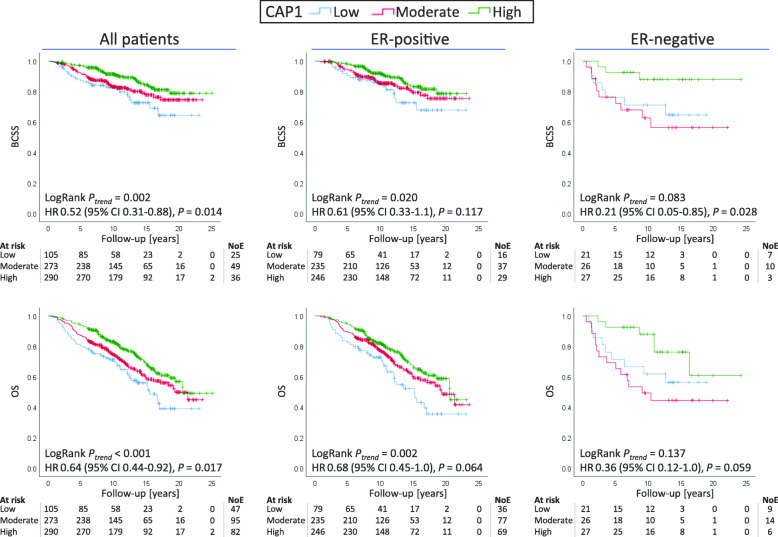
Predicted breast cancer-specific survival (BCSS) and overall survival (OS) comparing three groups of CAP1 cytoplasmic intensity. BCSS and OS among all patients and stratified by ER status. Patients at risk, number of events (NoE), LogRank trend test, and adjusted hazard ratios (HRs) with 95% CI comparing low CAP1 expression to high CAP1 expression are shown. HR adjusted for age at diagnosis (continuous), tumor size (> 20 mm, yes/no), and any axillary lymph node involvement (yes/no)

Patients with low CAP1 tumor expression had worse long-term survival, both in terms of BCSS (LogRank *P*_trend_= 0.002) and OS (LogRank *P*_trend_ < 0.001; Fig. 2), compared to the patients with tumors of moderate to high CAP1 expression. When stratified by ER status, the same trend was observed for patients with ER-positive breast cancer (BCSS: LogRank *P*_trend_ = 0.020 and OS: LogRank *P*_trend_ = 0.002; Fig. 2), whereas a non-linear association was found among patients with ER-negative breast cancer (Fig. 2).

### Survival differences by CAP1 expression in relation to body constitution

**Fig. 3:**
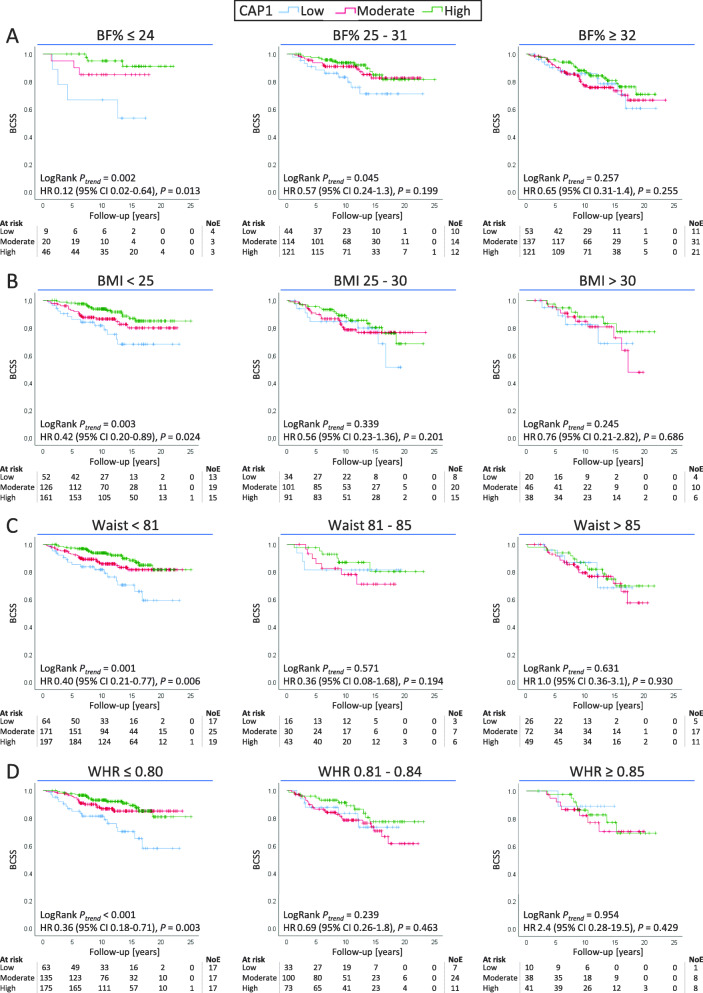
Breast cancer-specific survival (BCSS) according to CAP1 score, stratified for **a** body fat percentage, **b** BMI, **c** waist circumference, and **d** waist-hip ratio. Patients at risk, number of events (NoE), LogRank trend test, and adjusted hazard ratios (HRs) with 95% CI comparing low CAP1 expression to high CAP1 expression are shown. HR adjusted for age at diagnosis (continuous), tumor size (> 20 mm, yes/no), and any axillary lymph node involvement (yes/no)

In Kaplan-Meier estimates when patients were stratified on BF%, BMI, waist circumference, or WHR, CAP1 tumor expression was associated with worse BCSS among patients with low adiposity status across all four anthropometric measures, BF% (LogRank *P*_trend_ *=* 0.002; Fig. 3a), BMI (LogRank *P*_trend_ *=* 0.003; Fig. 3b), waist circumference (LogRank *P*_trend_ *=* 0.001; Fig. 3c), and WHR (LogRank *P*_trend_ < 0.001; Fig. 3d), compared with patients with higher CAP1 expression. No association between CAP1 expression and survival was observed for patients with the highest adiposity status. Similar results across anthropometric measures were found for the association between CAP1 expression and body fatness regarding OS (Additional file 4).

### CAP1 tumor expression and survival outcomes in adjusted models

**Fig. 4:**
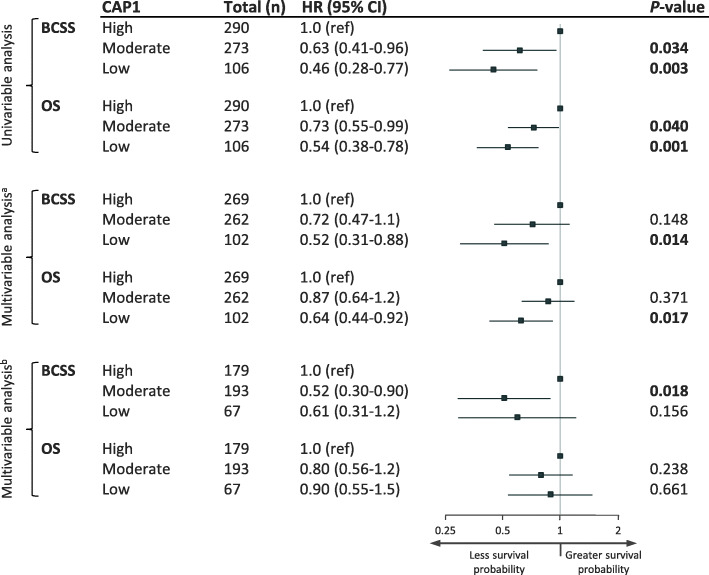
Breast cancer-specific survival (BCSS) and overall survival (OS) according to CAP1 expression. Forrest plot illustrating hazard ratio (HR) with 95% confidence interval (CI) across univariable analysis (model 1) and multivariable analyses (models 2 and 3). Model 2^a^ adjusted for age at diagnosis (continuous), tumor size (> 20 mm, yes/no) and any axillary lymph node involvement (yes/no). Model 3^b^ adjusted for as in Model 2^a^ along with histological grade III (yes/no), ER positive (yes/no), HER positive (yes/no) and Ki67 high (yes/no). *P*-value < 0.05 in bold

Univariable Cox analyses demonstrated that low CAP1 tumor expression was a prognostic indicator for inferior BCSS (HR = 0.46, 95% CI 0.28–0.77; Fig. 4) as well as poor OS (HR = 0.54, 95% CI 0.38–0.78; Fig. 4) among all patients. CAP1 remained a marker of poorer BCSS and OS after adjustments for age at diagnosis, tumor size, and any axillary lymph node involvement. However, the association did not remain after adjustments for histological grade, ER status, HER2, and Ki67. Among patients with ER-negative tumors and patients with low adiposity status, low CAP expression remained associated with poor BCSS after adjustments for age at diagnosis, tumor size, and any axillary lymph node involvement (Figs. 2 and 3). Further adjustment for BMI in the multivariable analyses did not significantly alter the results (data not shown).

## Discussion

In this prospective cohort study, the clinical impact of CAP1 tumor expression was evaluated in relation to body constitution and long-term survival outcomes in breast cancer. This assessment was performed following thorough antibody validation that ensured target specificity and functional application validity through a genetic strategy with siRNA-mediated *CAP1* knockdown. Two main findings were observed. First, low tumor-specific CAP1 protein expression was associated with anthropometric measures indicating a higher adiposity status and with unfavorable tumor characteristics linked to tumor aggressiveness. Second, patients with low tumor expression of CAP1 had an adverse breast cancer-specific and overall clinical outcome, with evidence of a stronger effect in lean patients.

Previous breast cancer studies have shown high *CAP1* gene expression to be associated with poor tumor characteristics and impaired relapse-free and overall survival [15, 20]. Overexpression of CAP1 has further been linked to poor prognosis in other types of cancer including lung cancer, hepatocellular carcinoma, and epithelial ovarian cancer [23, 25, 34]. Whereas *CAP1* gene overexpression consistently appears correlated to reduced cancer survival, few studies have reported the prognostic relevance for the corresponding protein level in breast tumor tissue.

In contrast to earlier gene expression studies of *CAP1*, low CAP1 tumor expression at the protein level was more frequent among patients with histologic grade III, high Ki67, or ER-negative tumors, indicating more aggressive tumors phenotypes. A reduced BCSS and OS for patients with low tumor-specific CAP1 protein expression compared with intermediate and high expression was observed. Low CAP1 remained an indicator of poor BCSS also in multivariable models adjusted for age at diagnosis, tumor size, and lymph node involvement. CAP1 expression was however not an independent prognostic factor for survival in the final multivariable model, which may partly relate to too small sample size with potential over adjustment in the fully adjusted model. Nonetheless, a relationship between lower CAP1 expression and decreased BCSS and OS remained across adjustments.

The discordant results regarding prognostic impact of *CAP1* gene expression and CAP1 protein expression needs to be considered. While gene expression data reflect the entire cellular compartment in the tumor tissue, immunohistochemical evaluations assess tumor cell-specific expression, which in part may account for conflicting results reported. Similar conflicting prognostic links between gene expression and tumor protein levels in breast cancer have been reported for the cell cycle regulator *CCND1* gene and corresponding cyclin D1 protein, as well as for PD-L1 gene and protein expression [35–37].

Two earlier breast cancer studies have assessed tumor CAP1 protein expression via IHC and report CAP1 positivity to be associated with unfavorable tumor characteristics (histologic grade III, ER-negative, lymph node positive) [38, 39]. High proportion of CAP1 positive tumor cells (> 30%) was prognostic of shorter OS [38]. The studies were limited by considerably smaller patient cohorts (*n* = 100), representing younger patients (median age approximately 50 years) with a remarkably higher proportion of ER-negative tumors (nearly 50%), compared with the large MDCS cohort reported herein. Variations in patient populations and analytical validity, including different antibodies and positivity thresholds used, likely explain the inconsistent results and impede direct comparisons.

Translational and posttranslational regulation of CAP1 are largely unknown, although phosphorylation of CAP1^S308/S310^ has been demonstrated to regulate its functional activity and control binding to the cytoskeletal proteins cofilin and actin, thus altering the cell migratory ability [40, 41]. One suggested phosphorylating agent for S310 is glycogen synthase kinase 3 [40]. CAP1 may further exert cell type-specific functions with a dual role regarding ER status. A decreased stimulation of cAMP, PKA, and NF-κB activity by resistin has been observed in CAP1 knockdown cells, indicating CAP1 is an upstream activator in inflammation, a process that may promote carcinogenesis [17]. Upon stratification for ER status, lower CAP1 expression remained associated with worse prognosis among patients with ER-positive tumors, while a non-linear trend was observed for ER-negative tumors. Depletion of CAP1 in experimental models stimulated proliferation, migration, invasion, and induction of epithelial-mesenchymal transition markers ERK and Snail in TNBC cells, while the adverse was observed in ER-positive cell lines [42].

Circulating levels of the adipokine resistin increase with adiposity and are associated with higher risk and impaired prognosis of breast cancer [16, 17]. Since CAP1 is the functional receptor to resistin, an additional aim of this study was to investigate CAP1 in relation to anthropometric measures. Lower CAP1 expression was positively associated with higher BMI, larger waist, wider hip, and higher BF%. These results indicate a relationship between obesity and lower CAP1 expression. The prognostic impact of CAP1 however appeared strongest among leaner patients as low CAP1 tumor expression was associated with reduced survival in patients with low adiposity status while no differences were found among patients with higher adiposity status.

There are a number of strengths to our study. First, this is the largest study of tumor-specific CAP1 expression of its kind allowing for patient stratification and multivariable analyses adjusted for relevant confounders. Second, this well-characterized prospective cohort with up to 25-year follow-up enabled long-term survival analyses relevant to the nature of breast cancer progression with late recurrences. Third, detailed anthropometric measures were taken by trained nurses, and not self-reported. Fourth, a genetic strategy was applied with siRNA-mediated CAP1 knockdown for thorough antibody validation to ensure target specificity and functional application validity. The main limitations are as follows: the immunohistochemical analyses were done on TMA, which does not represent a complete tumor tissue; however, it is an advantageous and commonly used method when analyzing material from large cohorts. Patients with no tumor tissue in the TMA were more likely to be older at baseline and presented with more favorable tumor characteristics where their tumors tended to be smaller, of lower grade, and node negative compared with patients with tumor tissue in the TMA. These differences thus indicate a potential selection bias towards patients with more unfavorable tumors characteristics in the final study population. In addition, breast cancer subtypes were constructed from surrogate classification and not intrinsic subtypes; information on anthropometric measures was collected at study entry (baseline) and not at time of breast cancer diagnosis. While this study is the largest to date, the final multivariate Cox analysis for all patients was still limited by small sample sizes that may affect the robustness of the test.

## Conclusions

In summary, our results demonstrate that lower CAP1 protein expression in early breast cancer was linked to higher adiposity status, more aggressive disease characteristics, and reduced long-term survival in women with breast cancer. These results highlight a contrasting link to earlier gene expression studies and differential prognostic information provided according to adiposity and ER status. The association of body constitution and breast cancer outcome is complex, and CAP1 is not working in isolation. CAP1 could be important as a predictor of poor prognosis in lean or ER-positive breast cancer patients, patient subgroups which generally have a favorable prognosis. This study demonstrates the importance to take body composition into consideration in clinical decision making. Additional large-scale studies are needed to fully elucidate the prognostic role of CAP1 in breast cancer and further investigate its clinical impact on breast cancer progression and disease recurrence.

## Supplementary information

**Additional file 1.** Validation of CAP1 antibody specificity and functional validity using a genetic approach of siRNA-mediated target knockdown. (A) Graphs displaying relative CAP1 mRNA expression in T47D and MDA-MB-231 cells following siRNA-induced CAP1 knockdown or non-silencing control (B) Quantification of relative CAP1 protein levels and Western immunoblotting showing reduction of protein bands detected by Abcam ab133655 at the expected molecular size (52 kDa) after CAP1 silencing, (C) Immunocytochemistry images visualizing CAP1 protein abundance in cell microarray of T47D and MDA-MB-231 in CAP1 knockdown or control cells. Error bars show standard error of means from three independent experiments.

**Additional file 2.** CAP1 protein expression grouped according to immunohistochemical staining. (A) Distribution over CAP1 scoring, (B) distribution over CAP1 scoring after grouping, (C) breast cancer-specific survival (BCSS) of all five different CAP1 scoring, (D) overall survival (OS) of all five different CAP1 scoring. Patients at risk and number of events (NoE) are shown.

**Additional file 3.** Distribution of breast cancer treatment and CAP1 tumor-specific expression.

**Additional file 4.** Overall survival (OS) according to CAP1 expression, stratified for (A) body fat percentage (BF%), (B) body mass index (BMI), (C) waist circumference, and (D) waist-hip ratio (WHR). Patients at risk, number of events (NoE), LogRank trend test and adjusted hazard ratios (HRs) with 95% CI comparing low CAP1 expression to high CAP1 expression are shown. HR adjusted for age at diagnosis (continuous), tumor size (> 20 mm, yes/no) and any axillary lymph node involvement (yes/no).

## Data Availability

The materials and data sets analyzed in the present study are available from the authors upon reasonable request. The data are not publicly available due to Swedish restrictions.

## Abbreviations

BF%: Body fat percentage
BMI: Body mass index
BCSS: Breast cancer-specific survival
CAP1: Adenylate cyclase-associated protein 1
CI: Confidence interval
ER: Estrogen receptor
HR: Hazard ratio
HER2: Human epidermal receptor 2
IHC: Immunohistochemistry
ISH: In situ hybridization
MDCS: The Malmö Diet and Cancer Study
OS: Overall survival
PR: Progesterone receptor
qRT-PCR: Quantitative reverse transcription PCR
siRNA: Small interfering RNA
TMA: Tissue microarray
