# Supplementary figures and images for: Effects of tumor-specific CAP1 expression and body constitution on clinical outcomes in patients with early breast cancer
Source: Breast Cancer Res. 2020 Jun 19;22:67. doi: 10.1186/s13058-020-01307-5 (PMC7304201; doi:10.1186/s13058-020-01307-5)

A

T47D

MDA-MB-231

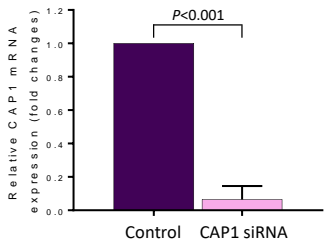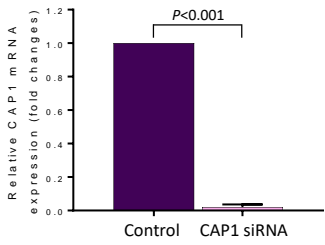

B

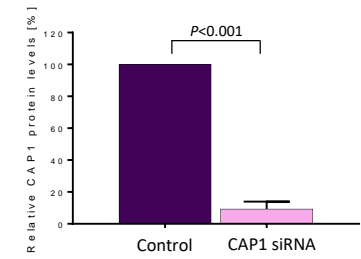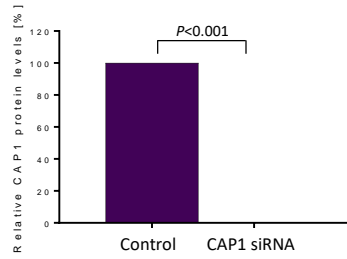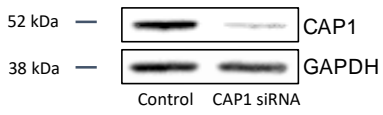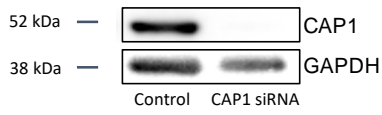

C

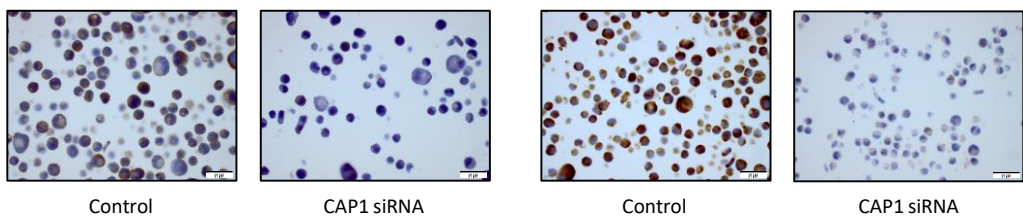

Supplement: Supplementary file 1 — Additional file 1. Validation of CAP1 antibody specificity and functional validity using a genetic approach of siRNA-mediated target knockdown. (A) Graphs displaying relative CAP1 mRNA expression in T47D and MDA-MB-231 cells following siRNA-induced CAP1 knockdown or non-silencing control (B) Quantification of relative CAP1 protein levels and Western immunoblotting showing reduction of protein bands detected by Abcam ab133655 at the expected molecular size (52 kDa) after CAP1 silencing, (C) Immunocytochemistry images visualizing CAP1 protein abundance in cell microarray of T47D and MDA-MB-231 in CAP1 knockdown or control cells. Error bars show standard error of means from three independent experiments. [file 13058_2020_1307_MOESM1_ESM.pdf]

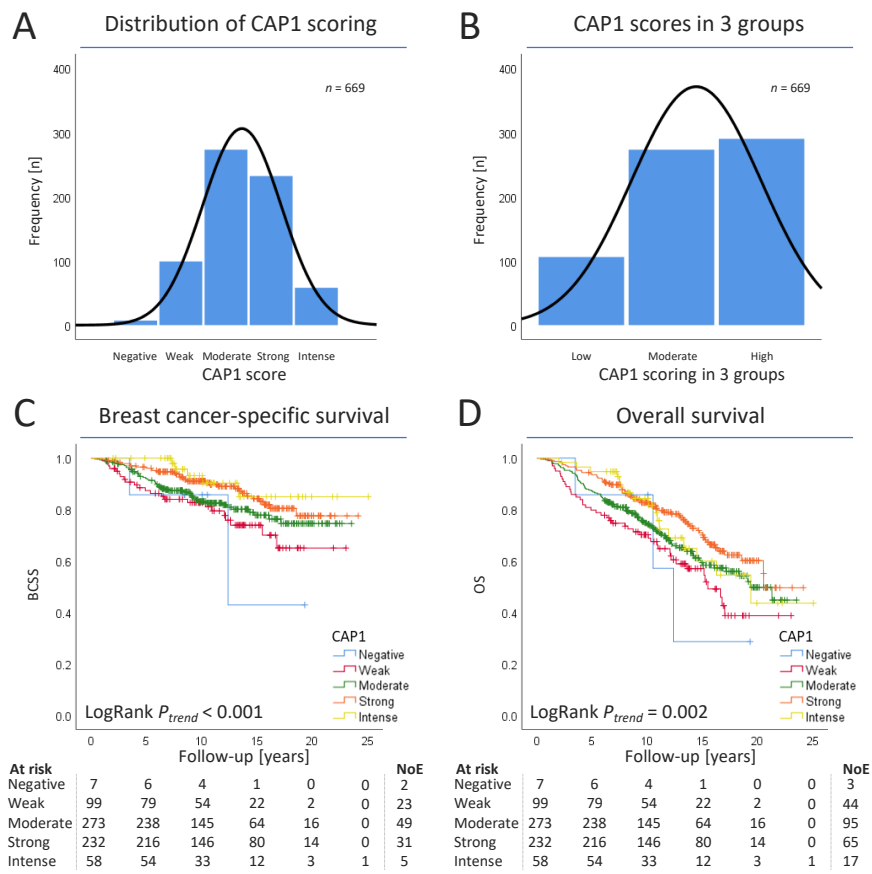

Supplement: Supplementary file 2 — Additional file 2. CAP1 protein expression grouped according to immunohistochemical staining. (A) Distribution over CAP1 scoring, (B) distribution over CAP1 scoring after grouping, (C) breast cancer-specific survival (BCSS) of all five different CAP1 scoring, (D) overall survival (OS) of all five different CAP1 scoring. Patients at risk and number of events (NoE) are shown. [file 13058_2020_1307_MOESM2_ESM.pdf]

CAP1 — Low — Moderate — High

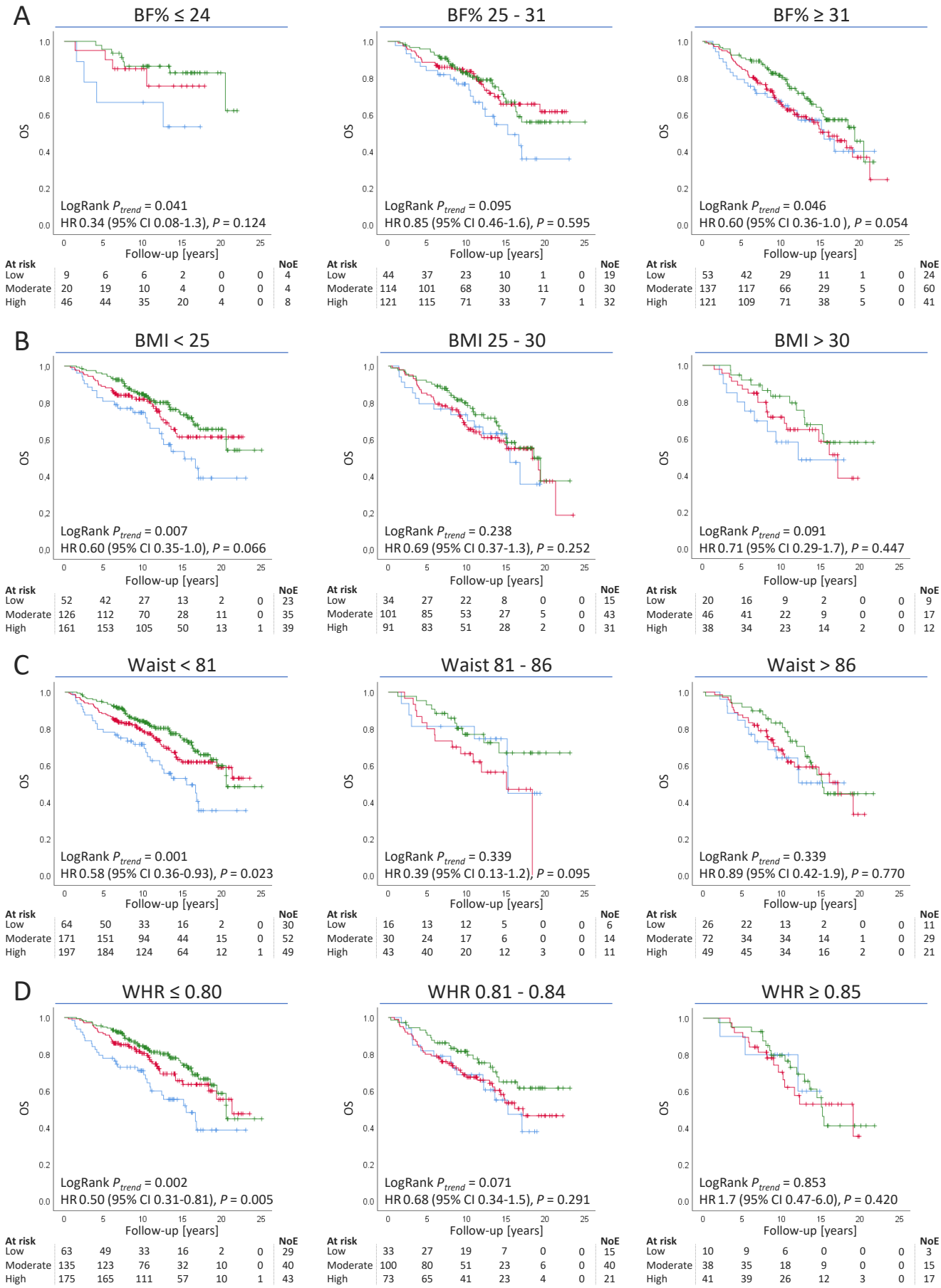

Supplement: Supplementary file 4 — Additional file 4. Overall survival (OS) according to CAP1 expression, stratified for (A) body fat percentage (BF%), (B) body mass index (BMI), (C) waist circumference, and (D) waist-hip ratio (WHR). Patients at risk, number of events (NoE), LogRank trend test and adjusted hazard ratios (HRs) with 95% CI comparing low CAP1 expression to high CAP1 expression are shown. HR adjusted for age at diagnosis (continuous), tumor size (> 20 mm, yes/no) and any axillary lymph node involvement (yes/no). [file 13058_2020_1307_MOESM4_ESM.pdf]
