# Supplementary material for: Effects of tumor-specific CAP1 expression and body constitution on clinical outcomes in patients with early breast cancer
Source: Breast Cancer Res. 2020 Jun 19;22:67. doi: 10.1186/s13058-020-01307-5 (PMC7304201; doi:10.1186/s13058-020-01307-5)
Supplement: Supplementary file 3 — Additional file 3. Distribution of breast cancer treatment and CAP1 tumor-specific expression. [file 13058_2020_1307_MOESM3_ESM.pdf]

Additional file 3.

Distribution of breast cancer treatment and CAP1 tumor-specific expression.

Patient characteristics<br>*n* (%) or median (IQR)	All patients<br>(*n*=910)	Patients included in TMA (*n*=718)<br>Low CAP1<br>(*n*=106)	Moderate CAP1<br>(*n*=273)	High CAP1<br>(*n*=290)	p_{trend}	Non-assessable<br>(*n*=49)	Not included in TMA<br>(*n*=192)
**Surgical procedure**							
Mastectomy	362 (41.7)	50 (49.0)	119 (45.4)	112 (39.3)		23 (47.9)	58 (33.7)
Lumpectomy	2 (0.2)	0 (0.0)	0 (0.0)	0 (0.0)		0 (0.0)	2 (1.2)
Partial mastectomy	504 (58.0)	52 (51.0)	142 (54.2)	173 (60.7)	0.060[b]	25 (52.1)	112 (65.1)
Biopsy only	1 (0.1)	0 (0.0)	1 (0.4)	0 (0.0)		0 (0.0)	0 (0.0)
Missing	41	3	10 (9+1)	4		1	20
**Surgical procedure, axilla**							
No, axillary dissection	79 (9.1)	5 (5.0)	10 (3.8)	20 (7.0)		4 (8.5)	40 (22.9)
Yes, axillary dissection	505 (58.0)	63 (62.4)	160 (61.1)	164 (57.5)	0.889[b]	32 (68.1)	86 (49.1)
Sentinel node	286 (32.9)	33 (32.7)	92 (35.1)	101 (35.4)		11 (23.4)	49 (28.0)
Missing	40	0	0	0		0	17
**Chemotherapy**							
Yes	126 (15.3)	21 (21.0)	47 (19.0)	39 (14.8)	0.115[b]	4 (8.9)	15 (9.0)
No	697 (84.7)	79 (79.0)	200 (81.0)	225 (85.2)		41 (91.1)	152 (91.0)
Missing	87	1	0	4		0	25
**Radiotherapy**							
Yes	499 (60.5)	62 (62.6)	160 (64.8)	165 (61.8)	0.723[b]	24 (53.3)	88 (52.7)
No	326 (39.5)	37 (37.4)	87 (35.2)	102 (38.2)		24 (46.7)	79 (47.3)
Missing	85	2	1	1		0	25
**Any endocrine therapy**							
None	408 (46.5)	32 (30.8)	97 (36.1)	132 (46.5)	**0.002[b]**	34 (69.4)	113 (65.7)
Yes	470 (53.5)	72 (69.2)	172 (63.9)	152 (53.5)		15 (30.6)	59 (34.3)
Missing	32	2	4	6		0	20

[a]Jonckheere-Terpstra test. [b]Linear-by-linear association test. *P*-value <0.05 in bold.
